# Effect of *Parthenium hysterophorus* L. Invasion on Soil Microbial Communities in the Yellow River Delta, China

**DOI:** 10.3390/microorganisms11010018

**Published:** 2022-12-21

**Authors:** Shuai Shang, Zaiwang Zhang, Liping Zhao, Longxiang Liu, Dongli Shi, Hui Xu, Hanjie Zhang, Wenjun Xie, Fengjuan Zhao, Zhihao Zhou, Jikun Xu, Jun Wang

**Affiliations:** 1 School of Biological and Environmental Engineering, Binzhou University, Binzhou 256600, China; 2 School of Environmental & Municipal Engineering, Qingdao University of Technology, Qingdao 266000, China; 3 Binzhou Shell Dike Island and Wetland National Nature Reserve Management Service Center, Binzhou 256600, China

**Keywords:** Alpha diversity, high-throughput sequencing, LEfSe analysis, rhizosphere soil

## Abstract

*Parthenium hysterophorus* L., as an invasive plant, has negatively impacted the ecosystem functioning and stability of the terrestrial ecosystem in China. However, little information was available for its effects on microorganisms in the Yellow River Delta (YRD), the biggest newly-formed wetland in China. In the present study, high-throughput sequencing technology was used to obtain the bacterial community in soils and roots of different plant species, including *P. hysterophorus* and some native ones in the YRD. Our results showed that the Proteobacteria, Acidobacteriota, Gemmatimonadota, and Actinobacteriota were dominant in the rhizosphere soils of *P. hysterophorus* (84.2%) and *Setaria viridis* (86.47%), and the bulk soils (80.7%). The Proteobacteria and Actinobacteriota were dominant within the root of *P. hysterophorus*. A total of 2468 bacterial OTUs were obtained from different groups among which 140 were observed in all the groups; 1019 OTUs were shared by *P. hysterophorus* non-rhizosphere soil bacteria (YNR) *P. hysterophorus* rhizosphere soil bacteria (YRR) groups. The indexes of the ACE (823.1), Chao1 (823.19), Simpson (0.9971), and Shannon (9.068) were the highest in the YRR groups, showing the greatest bacterial community diversity. Random forest analysis showed that the Methylomirabilota and Dadabacteria (at the phylum level) and the *Sphingomonas*, and *Woeseia* (at the genus level) were identified as the main predictors among different groups. The LEfSe results also showed the essential role of the Acidobacteriota in the YRR group. The SourceTracker analysis of the bacterial community of the YRR group was mainly from GBS groups (average 53.14%) and a small part was from YNR groups (average 6.56%), indicating that the *P. hysterophorus* invasion had a more significant effect on native plants’ rhizosphere microorganisms than soil microorganisms. Our observations could provide valuable information for understanding the bacterial diversity and structure of the soil to the invasion of *P. hysterophorus*.

## 1. Introduction

With the intensification of global environmental change, plant invasion has become an urgent problem and has attracted significant attention [1]. In China, coastal wetlands are faced with severe exotic species invasion problems. The Yellow River Delta (YRD) is a typical wetland ecosystem in China that supports high biodiversity [2] and has a very important ecological function. However, the invasive plant has negatively impacted the ecosystem functioning and stability of the YRD [3]. The invasive plants gradually replaced native plants with faster growth rates and more vital reproductive abilities, which further caused changes in soil physicochemical properties and microbial community structure, posing a severe threat to biodiversity and nutrient cycling in the YRD [4]. *P. hysterophorus*, native to America and the Gulf of Mexico, is an annual or short-lived perennial herb belonging to Asteraceae [5]. It is considered one of the worst weeds in the world; thus, once introduced to an alien environment, it can cause ecological and agricultural losses and serious environmental problems and damage to the biodiversity of native ecosystems [6]. It has a fast growth rate and solid reproductive ability, and its seeds are spread in various ways, leading to wide distribution as well as difficult removal [7]. In China, it was first identified in Yunnan Province. This weed was first found in Shandong Province in 2004, posing threats to the native plants and causing ecological and agricultural problems. Understanding the invasion mechanism of *P. hysterophorus* can help us to take appropriate control measures to protect the ecosystem and the biodiversity of the YRD.

Soil microorganisms are essential to wetland ecosystems [8]. They are sensitive to variations in biotic and abiotic factors and can be used as an indicator of ecosystem stability [9]. Changes in plant species and plant community diversity can affect soil microbial community structure and diversity [10]. Moreover, many studies have focused on the effect of soil properties on soil microorganisms. For example, soil pH significantly correlated with bacterial community structure [8]. Qu et al. reported that the low soil pH could inhibit soil bacterial enzymatic and metabolic activities, which hinders the growth of bacteria [11]. In addition, soil nutrients are considered an essential factor influencing soil microbial community diversity in wetlands.

Invasive plants can alter soil microbial community composition and diversity, creating a favorable soil environment and contributing to the success of their invasion [12]. Therefore, studying the influence of plant invasion on the soil microbial community can broaden our understanding of the effective mechanisms of invasive plants on wetland ecosystems. An increasing number of researchers have explored the response of soil microbial communities to invasive plants, such as *Spartina alterniflora* [2] and *Ageratina Adenophora* [12]. However, the response of soil microorganisms to invasive species *P. hysterophorus* has not yet been known. Different root exudates and litters of different invasive plants resulted in a heterogeneous soil microenvironment, which caused different soil microbial communities. Bulk soil, rhizosphere and root are important microhabitats that microorganisms inhabit and play different roles in the nutrient cycling of the wetland ecosystem. The different changes in these microhabitats due to the invasive plant may cause responses in the microbial communities residing at different microhabitats [12]. How the soil bacterial community residing at *P. hysterophorus* root, rhizosphere, bulk soil, and native plants differed in functional diversity is unknown. In this study, we selected three communities (*P. hysterophorus* community; a diverse community of *P. hysterophorus* and native plants; native plants community) in the YRD and explored their associated soil bacterial communities using high-throughput sequencing technology. By comparing bacterial community differences in invaded and non-invaded areas, this study can help to understand the response of soil bacterial diversity and structure to the invasion of *P. hysterophorus*. Moreover, it also provides a theoretical basis for invasive-plant control and wetland protection. In this study, we hypothesized that the bacterial community diversity and structure in the invaded area significantly differed from that in non-invaded areas.

## 2. Materials and Methods

### 2.1. Sampling Site

Soil samples were collected near Siyuan Lake, located in the Yellow River Delta (37°16′ N–38°16′ N, 118°20′ E–119°20′ E). The region has a warm temperate monsoon climate zone, with prominent continental meteorological characteristics and significant differences in the four seasons. The annual average maximum temperature is 18.3 °C, and the minimum temperature is 6.8 °C. The soil type is mainly saline soil, and the widely distributed vegetation includes *Suaeda salsa*, and *Tamarix chinensis* Lour. The sample plot is a plot of the *P. hysterophorus* seriously damaged, and the damage time is three years. The *P. hysterophorus* is a single population in the sample plot, and the density of the *P. hysterophorus* in the plot is 58, sporadically accompanied by *Setaria viridis (L.) Beauv*.

### 2.2. Sampling Collection, Determination of Soil Physical and Chemical Properties

In August 2022, *P. hysterophorus* rhizosphere soil, bulk soils, and *S.viridis* rhizosphere soils were collected in the study area. Five 1 m × 1 m quadrats with a distance of 20 m from the invasion plot were randomly selected, and five subsamples were collected in each quadrat using the 5-point sampling method. The five subsamples were mixed as the soil in the quadrat samples to reduce interference from soil heterogeneity. The soil adhering to the surface of the roots of *P. hysterophorus* by light brushing is the rhizosphere soil of each sub-sample. The rhizosphere soil of 5 sub-samples collected from each quadrat is thoroughly mixed to be the rhizosphere soil sample of 1 quadrat. If the rhizosphere soil collected from each quadrat is mixed, it is one rhizosphere soil sample. When the rhizosphere soil was collected, the bulk soil was also collected, and the bulk soil of the five plots was thoroughly mixed to obtain one bulk soil. The soil samples were divided into two parts. One part has been used to determine the soil’s physical and chemical properties. The other part was placed in a cooling box (temperature 4 °C) and immediately transferred to the laboratory −80 °C low-temperature refrigerator. Soil pH, electrical conductivity (EC) and the content of Total Organic Carbon (TOC) was determined in our previous studies [13].

### 2.3. High-Throughput Sequencing of Soil Microbe

The soil samples stored at −80 °C were divided into 1.5 mL centrifuge tubes in an ultra-clean workbench. The 4 replicates of each treatment resulted in a total of 16 soil samples. The root endophytes bacteria samples of *P. hysterophorus* were numbered Root group (Root1, Root2, Root3, Root4); the rhizosphere soil bacteria samples of *P. hysterophorus* were numbered YRR group (YRR1, YRR2, YRR3, YRR4); the bulk soils bacteria samples of *P. hysterophorus* were numbered YNR group (YNR1, YNR2, YNR3, YNR4); the soil bacteria samples of native plants were numbered GBS group (GBS1, GBS2, GBS3, GBS4). The V4 region of the 16S rRNA gene was amplified by PCR using 515F 5′~GTGCCAGCCGGTAA~3′ and 907R 5′~CCGTCAATTCTTRACTTT~3′. The PCR products of the same sample were mixed and then recovered with 2% agarose gel. The library was constructed using the Illumina NovaSeq for sequencing. The data that support the findings of this study are available in NCBI (https://www.ncbi.nlm.nih.gov/ (accession number PRJNA897086)) (accessed on 4 November 2022).

### 2.4. Data Analysis

The Alpha diversity index was calculated using Qiime software (Version 1.9.1) [14]. One-way analysis of variance (ANOVA) was used to determine the variances of soils properties among different groups. The PCoA graph was drawn by R (Version 3.4.4) [15]. The Alpha diversity index group differences and Beta diversity were calculated by Student’s t-test using SPSS (IBM SPSS Inc., Chicago, IL, USA) [16]. The Chao1 and Ace indices measure species abundance as the number of species. Shannon and Simpson’s indices are used to estimate species diversity, which is affected by species abundance and Community evenness in the sample community. The differences in bacterial community composition among different groups were identified by using LDA effect size (LEfSe). The difference in microbial community structure was tested using permutational multivariate analysis of variance (PERMANOVA). Significant differences were defined as *p* < 0.05. Random forest analyses were used to identify the essential dominant taxa in the non-rhizosphere soil for *P. hysterophorus* with the random forest package in R [17]. The SourceTracker was used to explore the composition proportion of sink samples from each Source [18].

## 3. Results

### 3.1. Soil Physical and Chemical Properties

In the present study, the pH was highest in the YNR group and lowest in the GBS group(Table 1). The pH was significantly different between the YNR and GBS groups (*p* < 0.05), while the pH was not entirely different between the YRR and YNR groups. The EC was highest in the YRR group, and lowest in the GBS group. The EC was not significantly different among different groups. The TOC content was highest in the YNR group, and lowest in the YRR group. The TOC content was significantly different between the YRR and YNR groups (*p* < 0.05), while the TOC content was not significantly different between the YNR and GBS groups.

### 3.2. Alpha Diversity Index Difference Analysis among Different Groups

**Figure 1:**
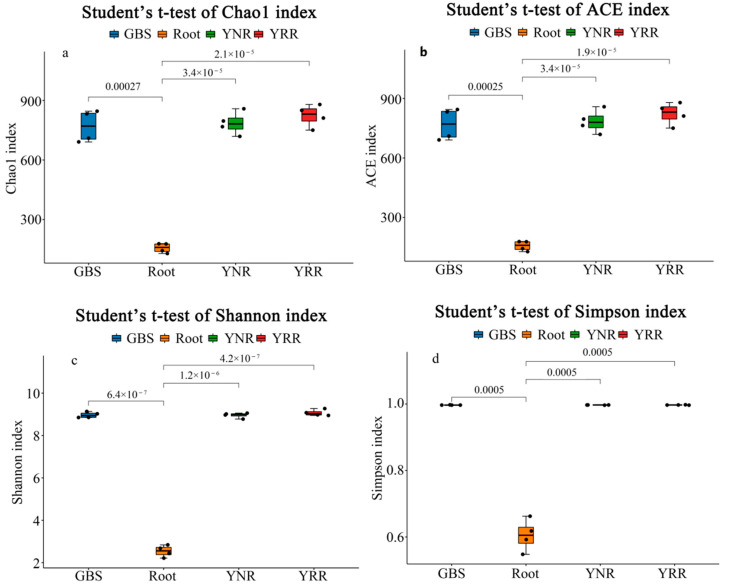
Alpha diversity indices of bacteria among different species. (**a**) Chao1; (**b**) ACE; (**c**) Shannon; (**d**) Simpson. Root: root endophytes bacteria samples of *P. hysterophorus*; YRR: the rhizosphere soil bacteria samples of *P. hysterophorus*; YNR: the bulk soils bacteria samples of *P. hysterophorus*; GBS: the soil bacteria samples of native plants were numbered GBS group.

Alpha diversity, including the species richness and diversity of single samples, was reflected by many measuring indexes, including Chao1, ACE, Shannon, Simpson, and coverage (Figure 1). Our results showed that the index of the ACE (823.1), Chao1 (823.19), Simpson (0.9971), and Shannon (9.068) are highest in the YRR groups, indicating the bacteria community diversity of the *P. hysterophorus* rhizosphere was higher in the rhizosphere than in other groups. The Chao1 index (156.68), ACE index (156.25), Simpson (0.605), and Shannon (2.543) are the lowest in root groups, which indicated that the abundance of root bacteria was lower than in other groups. The coverage ratios of all samples were more significant than 0.999, indicating that the sequences of samples were detected and indicating that the sequencing results could reflect the actual condition of the samples.

### 3.3. OTU Abundance Analysis

**Figure 2:**
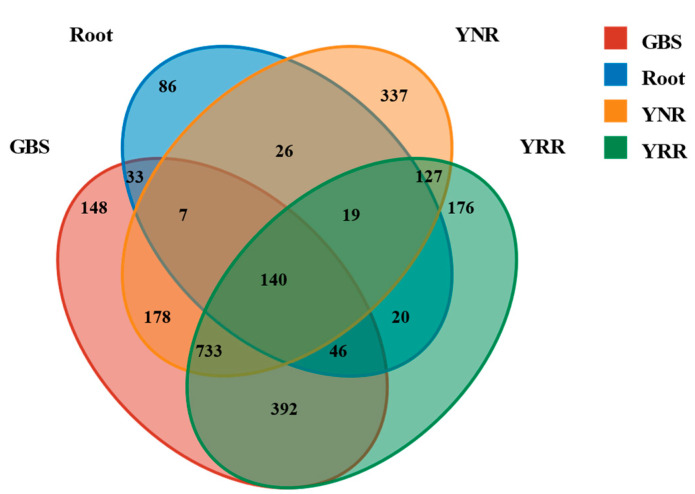
Venn diagram showing the number of shared and unique OTUs among different groups. Each circle represents sampled compartments. Values within intersections represent shared OTUs, and values outside intersections represent unique OTUs. Root: root endophytes bacteria samples of *P. hysterophorus*; YRR: the rhizosphere soil bacteria samples of *P. hysterophorus*; YNR: the bulk soils bacteria samples of *P. hysterophorus*; GBS: the soil bacteria samples of native plants were numbered GBS group.

In the present study, 1653 OTUs were identified in the YRR group; 1567 OTUs were identified in the YNR group; 1677 OTUs were identified in the GBS group; 377 OTUs were identified in the root group (Figure 2). A total of 2468 bacterial OTUs were obtained from different groups among which 140 were observed in all the groups; 1019 OTUs were shared by YNR and YRR groups; 1311 OTUs were shared by YRR and GBS groups; and 1083 OTUs were shared by YNR and GBS groups. The number of OTUs specific to rhizosphere and non-rhizosphere soil bacteria was higher than that of root bacteria.

### 3.4. Soil Microbial Community Structure Analysis

**Figure 3:**
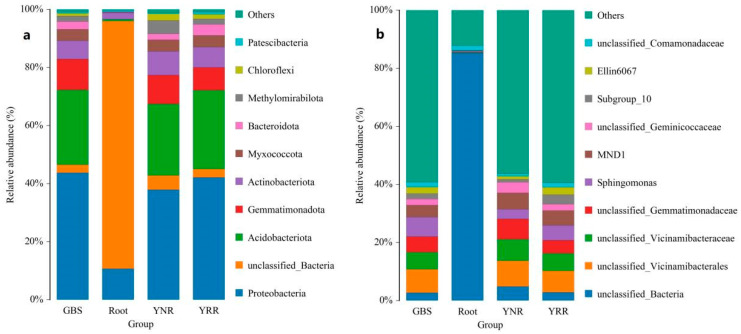
The relative abundance of the top 10 bacterial communities. (**a**) at the phylum levels; (**b**) at the genus levels. Root: root endophytes bacteria samples of *P. hysterophorus*; YRR: the rhizosphere soil bacteria samples of *P. hysterophorus*; YNR: the bulk soils bacteria samples of *P. hysterophorus*; GBS: the soil bacteria samples of native plants were numbered GBS group.

At the phylum level, the dominant bacteria in the rhizosphere and non-rhizosphere of *P. hysterophorus* were Proteobacteria, Acidobacteriota, Gemmatimonadota, and Actinobacteriota, and the total relative abundance was 84.2% and 80.7%, respectively. The dominant bacteria of *S. viridis* root soil were Proteobacteria, Acidobacteriota, Gemmatimonadota, and Actinobacteriota, and the total relative abundance of the four bacteria was 86.47%. The dominant bacteria within the *P. hysterophorus* root were Proteobacteria and Actinobacteriota, and the total relative abundance of the two bacteria in the rhizosphere of the *P. hysterophorus* root was 13% (Figure 3). At the genus level, except the unclassified species, the dominant bacteria were *Sphingomonas*, *MND1*, *Subgroup_10* and *Ellin6067* in the rhizosphere and non-rhizosphere of the *P. hysterophorus*, and the rhizosphere of the *S.viridis* (Figure 3).

### 3.5. PCoA Analysis

**Figure 4:**
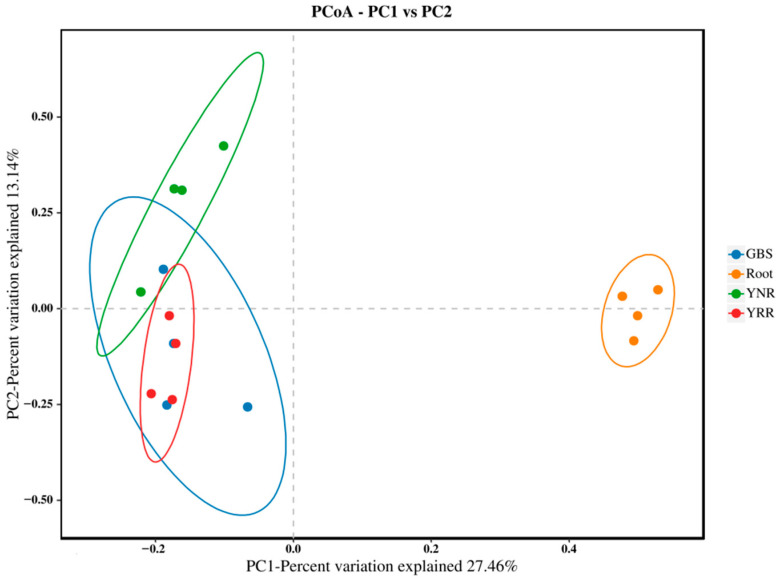
The PCoA based on the Bray–Curtis distance shows the variation in bacterial community structure. Different colors represent the samples from other groups. Root: root endophytes bacteria samples of *P. hysterophorus*; YRR: the rhizosphere soil bacteria samples of *P. hysterophorus*; YNR: the bulk soils bacteria samples of *P. hysterophorus*; GBS: the soil bacteria samples of native plants were numbered GBS group.

PCoA analysis can intuitively reflect the differences or similarities among different groups. Our results showed that the contribution rates of PC1 and PC2 were 27.46% and 13.14%, respectively (Figure 4). The distance between the bacterial communities in the rhizosphere soil of *P. hysterophorus* is relatively close, indicating their community composition is similar. In contrast, the root bacterial communities are more dispersed, meaning their community composition is quite different, and the community similarity is low. The comparison shows that the distribution distances of communities are quite different, indicating significant differences in the composition of the rhizosphere and root-soil bacterial communities of *P. hysterophorus*.

### 3.6. Correlation between Soil Bacterial Species and Soil Physicochemical Properties

**Figure 5:**
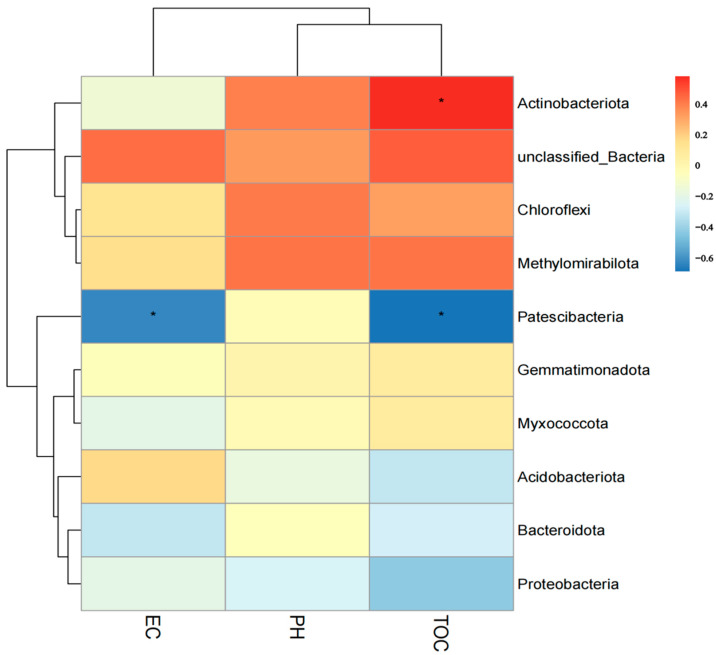
The relationship between the soil physicochemical properties and bacterial community. YRR: the rhizosphere soil bacteria samples of *P. hysterophorus*; YNR: the bulk soil bacteria samples of *P. hysterophorus*; GBS: the soil bacteria samples of native plants were numbered GBS group. * means significant at 0.05 level.

The results show that different environmental factors have other effects on soil microorganisms. The bacterial phyla in relative abundance and the soil physicochemical factors of pH, TOC, and EC were used as parameters, respectively. Our results showed that the Patescibacteria was correlated with the TOC and EC, and the Acitinobacteriota was correlated with the TOC (Figure 5).

**Figure 6:**
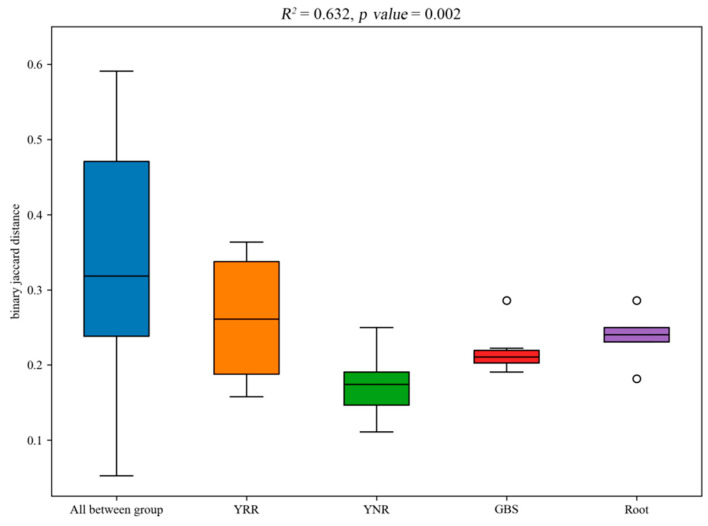
PERMANOVA analysis among different species. Root: root endophytes bacteria samples of *P. hysterophorus*; YRR: the rhizosphere soil bacteria samples of *P. hysterophorus*; YNR: the bulk soils bacteria samples of *P. hysterophorus*; GBS: the soil bacteria samples of native plants were numbered GBS group.

PERMANOVA is also called the multivariate analysis of variance, and it is mainly used in statistical approaches to analyze the similarity between multidimensional data sets. R^2^ obtained by PERMANOVA analysis represents the degree of explana tion of sample differences between different groups and the ratio of group variance to the total variance. Our results showed that the R^2^ was 0.632 (*p*-value = 0.002), indicating a higher degree of explanation of differences between groups and more significant group difference(Figure 6).

### 3.7. LEfSe Analysis and Random Forest Analyses

**Figure 7:**
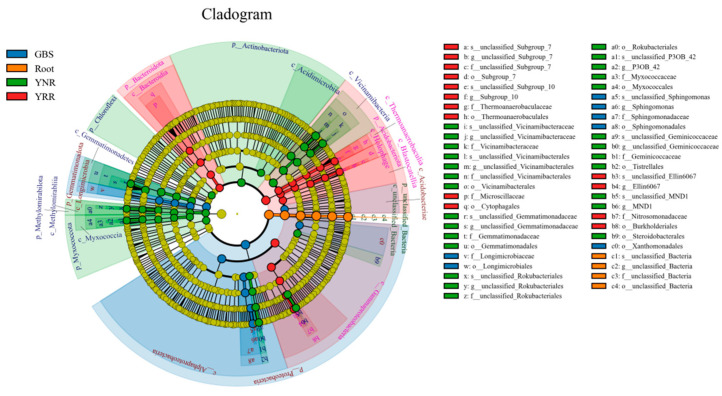
LEfSe analysis at different bacterial taxonomic levels among different groups. Different colored dots represent the taxa with significant differences among different samples. The inner to outer circles represent taxa from phylum to species. Root: root endophytes bacteria samples of *P. hysterophorus*; YRR: the rhizosphere soil bacteria samples of *P. hysterophorus*; YNR: the bulk soils bacteria samples of *P. hysterophorus*; GBS: the soil bacteria samples of native plants were numbered GBS group.

The LEfSe can find biomarkers with statistical differences between different groups. Our results showed that the Acidobacteriota and Bacteroidota (at the phylum level) and the *Subgroup_10* and *Ellin6067* (at the genus level) played essential roles in the YRR group (Figure 7). The Actinobacteriota, Methylomirabilota, Myxococcota and Chloroflexi (at the phylum level), and the *MND1* and *P3OB_42* (at the genus level) played an essential role in the YNR group. The Proteobacteria and Gemmatimonadota (at the phylum level), and the *Sphingomonas* (at the genus level) were playing an essential role in GBS groups.

**Figure 8:**
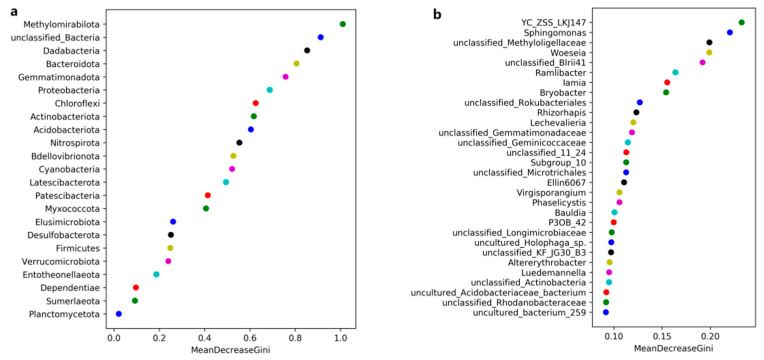
Random forest analyses among different species. Random forest is a subclass of ensemble learning, which depends on the voting choices of decision trees to determine the final classification results. Different colors mean different bacterial species. (a) at the phylum levels; (b) at the genus levels.

Random forest analyses were conducted to identify the most critical dominant taxa in the rhizosphere (Figure 8). Our results found that the Methylomirabilota and Dadabacteria (at the phylum level), the *Sphingomonas* and *Woeseia* (at the genus level) were identified as the main predictors among different groups.

### 3.8. SourceTracker Analysis

**Figure 9:**
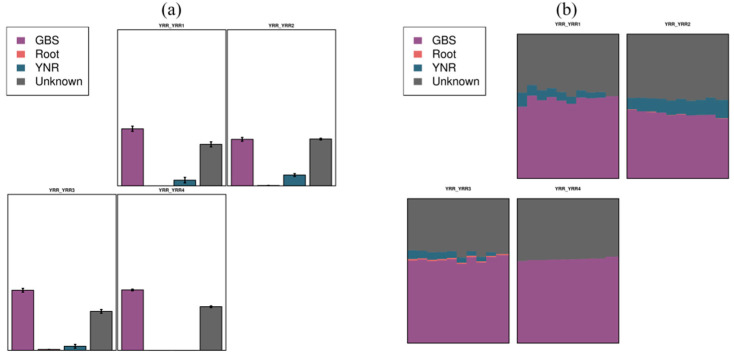
SourceTracker analysis among different species. (**a**) graph represents a predicted sample, with different colored columns representing the proportions of each source in that sample. Unknow represents the unknown source classification; (**b**) proportional area map of predicted sample sources. One graph represents a prediction sample; different colors represent the proportion of different sources.

In the present study, the YRR group was identified as sink samples, and the GBS and YNR groups were identified as the source. Based on the Bayesian algorithm, the analysis of sources in the target samples was explored. The composition proportion of sink samples from each source sample was predicted according to the community structure distribution of source samples and sink samples (Figure 9). In the present study, the YRR group was identified as sink samples. Our results showed that the bacterial community of the YRR group was mainly from GBS groups (average 53.14%), a small part from YNR groups (average 6.56%), and the unknown source was an average of 40.3%.

## 4. Discussion

The soil microbial community composition is closely related to the plant community. Plant invasion often leads to severe changes in plant community composition, resulting in the chemical composition of root secretion and litter change [19]. Eventually, the corresponding succession of soil microbial community structure and the shift in soil microbial community is conducive to the further invasion of invasive plants [20]. The rising trend of plant invasion, the invasion of the ecological environment, social economy, ecological security, and one kind of health had a severe effect; exotic plant invasion has become a problem to be solved [20]. One of the essential effects of plants on the soil environment is to change the characteristics of soil microbial communities. In the invasion process, invasive plants can change the soil microbial diversity and community structure and function of the invaded area, thereby enhancing their adaptability to the environment and benefiting their invasion and growth [21]. The change of soil microorganisms also reacts to the soil environment. By changing the physical and chemical properties and physical properties of the soil, it is more suitable for the colonization and expansion of invasive plants. Previous studies found that the invasion of *Solidago canadensis* depends largely on beneficial microorganisms in the rhizosphere, which is also closely related to its competitiveness [22].

We analyzed the composition of the *P. hysterophorus*’s root, rhizosphere, non-rhizosphere, and native plant microorganisms in the present study. The common bacteria were Proteobacteria and Actinobacteriota at the phylum level. Random forest research showed that the microbial composition showed a downward trend from the outside to the inside. We speculated that *P. hysterophorus* would benefit its microorganisms from the surrounding environment, thereby participating in its metabolic activities and capacity absorption. Previous studies found that the Bacterial endophytes played an essential role in resistance or tolerance to the host plant from biotic and abiotic stresses [23]. Our results showed that only 377 OTUs were identified in the Root group, and the bacterial community of root endophytes was significantly different from other groups. We speculated that the root endophytes of *P. hysterophorus* have a particular specificity. Previous studies showed that Acidobacteriota was well-adapted to low-nutrient environments [24], representing a highly diverse phylum resident to a wide range of habitats around the globe [25]. In addition, Acidobacteriota was involved in using nitrite as the N source, responding to the soil, expressing multiple active transporters, degrading gellan gum, and producing exopolysaccharides [25]. In our study, the LEfSe results also show an essential role of the Acidobacteriota in the YRR group. Thus, we speculated that the Acidobacteriota was necessary for the *P. hysterophorus* invasion.

The soil microbial community can form a relatively cooperative and stable ecological relationship with native plants in a specific growth environment. However, the soil microbial community is also volatile and will be affected by invasive plants and plant community diversity changes [26]. There are differences in microbial diversity and richness between *P. hysterophorus* rhizosphere soil and non-rhizosphere soil. The ACE (823.1), Chao1 (823.19), Simpson (0.9971), and Shannon (9.068) index of the YRR groups are higher than that of the YNR groups, which indicates a higher community diversity and richness in *P. hysterophorus* rhizosphere. We speculated that invasive plants could change the structure and reduce the diversity of the soil microbial community in the invasion site.

Once there is exotic plant invasion success in the new habitat, under the condition of being suitable, it will spread diffusion, and the soil microbial community changes with the change of the plant communities on the ground [27]. With an in-depth understanding of the underground part of the ecological system, most scholars believe that invasive plants can, directly or indirectly, alter the soil microbial community [28]. The SourceTracker analysis showed that the bacteria of the YRR groups were mainly from the GBS groups, and only a tiny part was from the YNR group. Meanwhile, 1311 OTUs were shared by YRR and GBS groups. The bacteria community of the *P. hysterophorus* rhizosphere was more similar to the *S. viridis* rhizosphere than the *P. hysterophorus* non-rhizosphere. We speculated that *P. hysterophorus* could significantly affect the bacterial community of the native plant rhizosphere. The impact of invasion on soil bacterial community characteristics shows that changing soil bacterial community may be an essential part of the invasion process of *Eurasian japonica*. The diversity of bacteria in rhizosphere soil is higher than that in non-rhizosphere soil, and the richness is lower than that in non-rhizosphere soil. Previous studies found that for invasive plants in the process of invasion to land with soil microbial diversity, community structure and functional changes enhance their ability to adapt to the environment, which are conducive to the plants’ invasion and growth [21].

## Figures and Tables

**Table 1:** Changes of soil physicochemical properties among groups.

Groups	EC (ms/cm)	pH	TOC (g/kg)
YRR	2.16 ± 0.56a	7.78 ± 0.07ab	18.33 ± 0.90b
YNR	2.14 ± 0.19a	7.90 ± 0.07a	47.08 ± 7.41a
GBS	1.87 ± 0.24a	7.69 ± 0.02b	25.74 ± 7.68ab

TOC means Total Organic Carbon, and EC means electrical conductivity. Values are the means ± standard error (n = 4). Lowercase letters in the same column indicate significant differences between the two groups (*p* < 0.05). YRR: the rhizosphere soil bacteria samples of *P. hysterophorus*; YNR: the bulk soil bacteria samples of *P. hysterophorus*; GBS: the soil bacteria samples of native plants were numbered GBS group.

## Data Availability

The data that support the findings of this study are available at NCBI (https://www.ncbi.nlm.nih.gov/ (accession number PRJNA897086)) (accessed on 4 November 2022).
